# Nursing & Midwifery students’ experience of immersive virtual reality storytelling: an evaluative study

**DOI:** 10.1186/s12912-020-00471-5

**Published:** 2020-08-17

**Authors:** Philip Hardie, Andrew Darley, Lorraine Carroll, Catherine Redmond, Abraham Campbell, Suzi Jarvis

**Affiliations:** 1 School of Nursing, Midwifery and Health Systems, University College Dublin, Dublin, Ireland; 2 School of Computer Science, University College Dublin, Dublin, Ireland; 3 Innovation Academy, University College Dublin, Dublin, Ireland

**Keywords:** Virtual reality, Immersive virtual reality storytelling, Nursing and midwifery education

## Abstract

**Background:**

Immersive Virtual Reality (iVR) storytelling is a concept that merges ground-breaking virtual reality technology with the traditional art of storytelling. Virtual reality storytelling offers a rare opportunity to present abstract experiences that challenge boundaries, heighten emotions, and convey previously intangible concepts. Scientific research into immersive virtual reality storytelling is still in its infancy, particularly regarding the field of education in Nursing and Midwifery. Therefore, this study set out to investigate the subjective experience of using an immersive virtual reality storytelling experience as an active pedagogy.

**Methods:**

This was an evaluative study incorporating a multimodal approach encompassing a cross-sectional survey and observational study conducted in a large University in Ireland, offering major undergraduate and graduate degree programmes in the fields of Nursing and Midwifery. Students were invited to view the innovative virtual reality storytelling experience “Wonderful You” (BHD Immersive) that tells the story of the first 9 months of a baby’s life inside the woman’s womb. On completion, students were asked to complete an anonymous survey about their experience. Observational studies were also carried out, examining the student’s engagement and interaction with the iVR experience. A combination of statistical and thematic qualitative analysis was employed to interpret the respective summative rating scale and open-ended response questions in the evaluation survey. Data captured from the observations were grouped into categories and analysed capturing key themes.

**Results:**

A response rate of 71.2% (*n* = 94) identified iVR storytelling as a memorable learning experience that triggered students’ engagement and motivation to learn. IVR storytelling enabled students to visualise and better understand abstract concepts. Qualitative analysis of narrative responses revealed the positive evaluations of the iVR storytelling experience. Observational studies further revealed students were highly engaged and interacted positively with the iVR storytelling experience.

**Conclusions:**

The full potential of this new medium of iVR storytelling has yet to be seen. However, this study provides an encouraging insight into the positive attributes of iVR storytelling that engages students and creates authentic active learning experiences.

## Background

Virtual reality (VR) is a technology that continues to grow in popularity. Advances in VR technology have led to the potential use of VR in Nursing and Midwifery education to create innovative and memorable learning opportunities. The term “Virtual Reality” was popularised in 1988 by Jaron Lanier, American philosopher, and computer scientist through his company VPL Research [1, 2]. VR is defined as “*a three-dimensional simulated environment based on technology that enables the user to interact with real-world simulations or alternative realities*” [3]. Lioce et al. [4] further define VR as “*a computer-generated three-dimensional environment that gives an immersion effect”*. VR permits users to view content through a head-mounted display (HMD). A stereoscopic lens which permits a three-dimensional effect is used in the HMD to immerse the user into the experience.

The application of VR as a pedagogical approach adheres to constructivist learning theory and speaks to the paradigm of experiential learning. In line with constructivist learning theory, students take an active role in their learning through VR; absorbing new information in the VR experience but also connecting it to previous knowledge to construct new knowledge [5]. Experiential learning theory suggests that immersive technology can enhance learning performance by providing learners with meaningful experiences [6]. The theory framework conveys learning as a continuous process that moves in four stages and Huang et al. [7] advocates implementing the four stages to support active learning through VR. Stage 1 consists of the concrete experience: involving participation in a VR experience. Stage 2 involves observation and reflection, e.g., debriefing and identifying areas of strengths and weakness throughout the VR experience and identifying any discrepancies between the experience and understanding of a concept in the VR experience. Stage 3 concerns formation of abstract concepts, e.g. what was learned from the VR experience and feedback received from a facilitator. Stage 4 involves testing of new situations, e.g. planning or practically applying the learning to future situations [7].

As early as 2002 Stapleton et al. [8] identified imagination as one of the key features of VR. Burdea & Coiffet [9] further determined an additional two core features of VR creating the i3 triangle “Immersion, interaction and imagination”. Immersion refers to the physical level of submersion in the VR experience and is determined by the degree a user associates themselves in a simulated virtual world [10], with Nilsson et al. [11] arguing the level of immersion is a result of the affordance of VR technology e.g. High End VR facilitates a greater level of immersion. The feeling of “being immersed” is subjective and generally associated with the emotional capacity of a person. It is the feeling of being involved with an experience [12]. The second feature of VR is its level of interaction, defined as the degree of accuracy and responsiveness a user’s actions represent in the VR world when using VR input hardware such as motion sensor gloves [10]. Finally, VR is grounded with the user’s imagination and refers to the mind’s capacity to perceive non-existent things in the VR world whether it be objects or a complete alternative universe, despite knowing they are physically situated in another environment [13]. Huang et al. [5] believes that with increased immersion, interaction, and imagination that VR provides, students’ motivation for learning increases. VR immerses students in a place or situation that prompts the exploration of new concepts, therefore, enhancing problem-solving capabilities through active discovery.

More specifically, Immersive Virtual Reality (iVR) Storytelling is a concept that combines the emerging world of VR technology and the art form of classical storytelling. iVR storytelling involves a user-focused engagement with the unfolding narrative either as a witness or participant [14]. This new format of storytelling differs from traditional narrative media, such as film and tv in which the viewer, for the most part, is a passive participant. Storytelling in VR is less about telling the viewer a story and more letting the viewer discover the story [15]. Prof. Uri Hasson’s exploration of the science of storytelling has revealed that stories activate multiple senses in the brain; motor, auditory, olfactory, somatosensory, and visual [16]. The triggering of these senses can help the listener/viewer of the story to understand the essence of complex concepts and ideas in a personal and meaningful way [17]. Stories can create memorable experiences that evoke emotions, making it easier to recall due to the power of their sensory associations [18]. In essence, stories that are personal and emotionally compelling engage the brain and are therefore better remembered than merely stating facts. IVR storytelling provides a unique opportunity to present abstract experiences that push boundaries, stir emotion and express concepts that were previously intangible [15].

The application of traditional storytelling is widespread in nurse education, with lecturers discussing stories from their clinical practice or other experiences to promote student learning and transfer practical knowledge gained through experience to the novice student [19]. Storytelling engages the students, contextualises theory to practice, can role model good practice and can help students envisage a life as a nurse [20]. Moreau et al’s [21]. systematic review on digital storytelling in healthcare education describes students’ self-reported positive learning experiences and in some cases changes in behaviour as a result of a digital storytelling experience by healthcare professionals. The tool of digital storytelling was observed as effective in developing skills and motivation in students’ learning [21].

The use of conventional multimedia-based learning such as digital storytelling via a desktop and web-based applications are now further extended by iVR, as it can facilitate a deeply engaging learning environment. While some research has been carried out on examining traditional/digital storytelling, no empirical studies have reported the application of iVR storytelling in Nursing and Midwifery education in the literature to date.

Current applications of iVR in nursing education include anatomy education [22–24], development of technical and procedural skills, including urinary catheterisation [25], care of the older adult [26, 27] and interprofessional and communication skills development [28]. Scientific research into the field of iVR storytelling is still in its infancy, particularly regarding the area of Nursing and Midwifery education. Therefore, the current study set out to explore Nursing and Midwifery students’ perceived experience of using an iVR storytelling experience as an active pedagogy.

The aims of the study were to explore:

- Students’ perception of an immersive VR storytelling as an active pedagogy
- Students’ perception of levels of immersion, interaction, imagination, and motivation during an experience of immersive VR storytelling
- Students’ engagement and interaction with immersive VR storytelling

## Methods

### Design and setting

This was an evaluative study incorporating a mixed-method design consisting of a cross-sectional survey and an observational study. This study was conducted in a large University in Ireland, offering major undergraduate and graduate degree programmes across Nursing and Midwifery.

### Sample and procedure

Students from Bachelor of Science (BSc) and Postgraduate (Higher Diploma) in Children’s and General Nursing and Midwifery programmes were invited to participate. All students had previously received traditional lectures on embryology, however the development of the five senses encountered in the iVR experience was a new concept to all students. Following a lecture students were informed about the proposed study and invited to participate. Participants were informed their participation was entirely voluntary, and that the iVR storytelling experience was an optional supplementary educational experience to their designated curriculum. Participants were also informed that their coursework or related grades would not be impacted in any way if they chose not to participate. An information leaflet was provided to potential participants describing the study and scheduled times the iVR experience would be available in the simulation laboratories on the university campus if they chose to participate. Interested students voluntarily booked a set date and time that suited them. Written consent to participate in the study was received on arrival to the simulation laboratory. Following the completion of the iVR storytelling experience, students were then asked by the researchers to complete an online anonymous questionnaire. Student confidentiality and privacy was maintained throughout the study through the following steps; no identifiable information was recorded within the online questionnaire, no record of student names was recorded in the observational studies and the consent forms were only held by the lead researcher and locked in a secure filing cabinet. A total of 132 students were invited to take part in the study. Ninety-four students completed the iVR experience and completed the questionnaire, resulting in a 71.2% response rate. An ethical exemption was granted by the University ethics committee on the grounds of the study being an educational evaluation.

### Investigation tool and methods

This study utilised the award-winning open access iVR experience “Wonderful You” (https://www.wonderfulyouvr.com/), developed by BDH Immersive in collaboration with Dr. David Barker, a developmental biologist in the United Kingdom. It tells the story of the first 9 months of a baby’s life inside the mother’s womb; from a tiny cluster of cells to a fully formed baby, focusing on the formation and development of the five senses (sight, sound, touch, taste and smell). To evaluate students’ attitudes towards iVR storytelling the well validated Huang et al. [5] questionnaire was adapted with permission. Huang et al. [5] tool was developed to investigate learners’ attitudes toward virtual reality learning environments (VRLE) and is based on a constructivist theoretical approach, that examines student’s interaction, immersion, imagination, motivation, and problem–solving capability with VRLE. For this study, the research team added a set of questions on immersive VR storytelling. The adapted questionnaire contained both summative rating scale response options that utilised a 5-point Likert scale (strongly disagree (1) and strongly agree (5) and open-ended responses such as “*What did you like most about using immersive virtual reality storytelling (via a headset) in the classroom*?”. Testing of the adapted tool revealed a Cronbach 훼 coefficient > 0.7 for each item. Internal consistency for the questionnaire was α = 0.95. An internal consistency reliability test of the original questionnaire carried out by Huang [5] also reported the alpha reliability was highly accepted (α =0.94). Further details of the instrument will be covered in the analysis section. The questionnaire was offered to students using a web-based survey service after exposure to the iVR storytelling experience.

Participant observation was also conducted throughout the iVR storytelling experience by a member of the research team in each simulation laboratory where the event took place. Typically, 12–15 participants were accommodated per room. The researcher was situated at the top of the room to enable a clear viewpoint to observe all participants. Each researcher was provided with a semi-structured observation guide based on observational principles outlined by Menter et al., [29] for examining educational practices. They were directed to observe and note the student’s engagement with VR and their interaction with the iVR storytelling experience e.g. did the students have any difficulties setting up the VR headset, or how did the students interact with the iVR experience for example their actions and body language. The semi-structured observation guide facilitates the recording of behavioural patterns and interactions but also permits flexibility to record comments and unexpected outcomes. Data gathered through participant observation was used as a benchmark against participants’ subjective reporting in the questionnaire of their perceptions and interactions with VR. A short demonstration on the setup of their phone and VR headset was provided at the start of each session. Participants were asked to download a free third-party VR app via Google Store or iTunes Store; Within (https://www.with.in/). Once downloaded the iVR experience “Wonderful You” could be accessed free of charge from the library of available iVR experiences. Each participant was provided by the University a Head Mounted Display (HMD) (VR One Plus Mobile Virtual Reality headset; est. cost per headset €49). The HMD delivers high optics through precision eyeglasses lenses which supports a wide range of interpupillary distance 53-77 mm without the need for manual adjustment by users, therefore making the HMD suitable for participants who may wear glasses also. Participants removed the universal tray from the HMD headset and placed their personal smartphones in the tray (which supports smartphone displays sizes ranging from 4.7 up to 5.5 in.). It is important to note the smartphone must have an accelerometer, gyroscope, and compass for the iVR application to work. Once fitted in the tray, students inserted the tray into the front of the HMD. At this point most students (*n* = 62) utilised their personal audio headphones via the audio port in their smartphone or via Bluetooth. The remaining students did not bring their personal audio headphones and were therefore advised by the facilitator to adjust the volume on their smartphone so that it was both audible to them and limited distraction to other students. Instructions were provided to switch the phone to Aeroplane/Flight Mode to avoid distractions during the experience such as text messages or app notifications. Participants were guided by researchers on adjusting the head strap of the HMD for comfort purposes. Participants were reminded that the VR experience is a 360-degree experience, and to be mindful of their surroundings when positioning themselves to view the iVR experience. Students sat dispersed throughout the simulation lab, approx. 8 to 10 m apart with some sitting on chairs, others sitting on the beds in the room. Researchers were instructed to assist the students with any technical difficulties setting up but not to interrupt the students once the iVR experience had begun.

### Data analysis

Quantitative data analysis was performed using the Statistical Package for Social Sciences (SPSS version 24). Descriptive data analysis was utilised to examine the frequency distribution of each item based on student responses to the statements of the questionnaire. The mean (SD) rating score of each item was also calculated. Qualitative data were collected via open-ended responses within the survey and observational studies during the VR experience. Thematic analysis was applied using Braun & Clarke’s [30] six-phase framework: Step 1: Familiarization, Step 2: Coding, Step 3: Generating themes, Step 4: Reviewing themes, Step 5: Defining and naming themes, Step 6: Writing up. Unlike many qualitative methodologies, thematic analysis is a method that is not affiliated to a specific epistemological or theoretical perspective, therefore making it a flexible method suitable for investigating teaching and learning approaches that are incredibly diverse in nature Maguire & Delahunt [31]. The researchers applied a top-down approach by identify key themes and selecting quotations to the illustrate the key findings to address the research questions. Data from the observational studies were transferred to Excel, were grouped into categories and analysed capturing key themes. Two researchers coded separately to enhance the validity of both processes (P.H./L.C Quantitative, P.H./A.D. Qualitative/Observations). As data was anonymous, data emerging from the observation sessions were not linked to individual participants, rather the observation data was used to triangulate and contextualise the quantitative and qualitative findings. The raw datasets used and/or analysed are available from the corresponding author upon reasonable request.

## Results

### Demographic results

The demographic profile of the participants is presented in Table 1 below. Most participants were Irish (93.46%), female (98.9%) and aged between 18 to 24 (66%). 55.3% (*n* = 53) participants were undertaking a Midwifery programme and 44.7% (*n* = 43) were undertaking a Children’s Nursing programme.

**Table 1:** Demographic profile of the sample

	n = 94	
	n	(%)
**Age (years)**		
18–24	62	(66)
25–34	27	(28.7)
35–44	4	(4.3)
≥ 45	1	(1.1)
**Gender**		
Female	93	(98.9)
Male	1	(1.1)
**Nationality**		
Irish	88	(93.6)
European	5	(5.3)
Non-European	1	(1.1)
**Programme**		
Midwifery	52	(55.3)
Children’s Nursing	42	(44.7)

### Questionnaire results

**Fig. 1:**
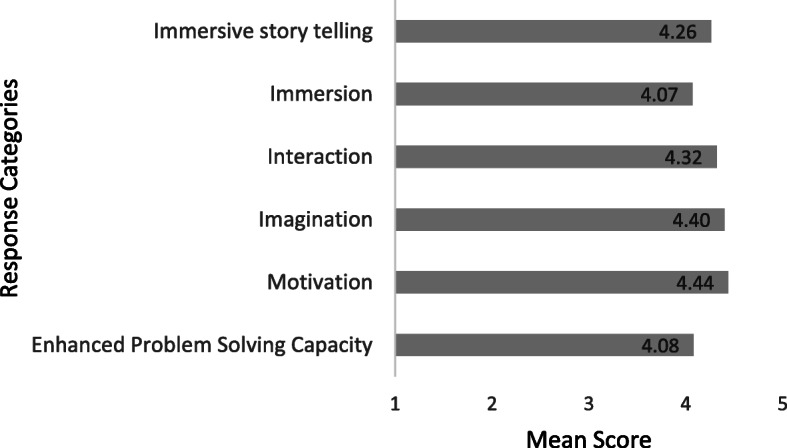
Mean score for each subsection of the questionnaire (*n* = 94). Score 1 = Strongly Disagree; 2 = Disagree; 3 = Neither Agree nor Disagree; 4 = Agree; 5 = Strongly Agree

The overall mean score of the questionnaire was 4.26 (SD 0.52; range 1–5), indicating that participants reported positively to engaging with and learning from an iVR storytelling experience. Figure 1 below summarises the mean score for each of the subscale themes. All items on the instrument were rated between strongly disagree (1) and strongly agree (5). The greatest perceived gain from implementing iVR storytelling was students’ motivation, which received an overall mean score of 4.44.

To further explore which item in each subscale contributed to participants learning, each item response was examined. Table 2 below outlines the percentage of scores in each of the 6 categories for all 28 items grouped under their respective subscales. The final column provides the mean (SD) rating score of each item illustrating those with the most and least impact .

**Table 2:**
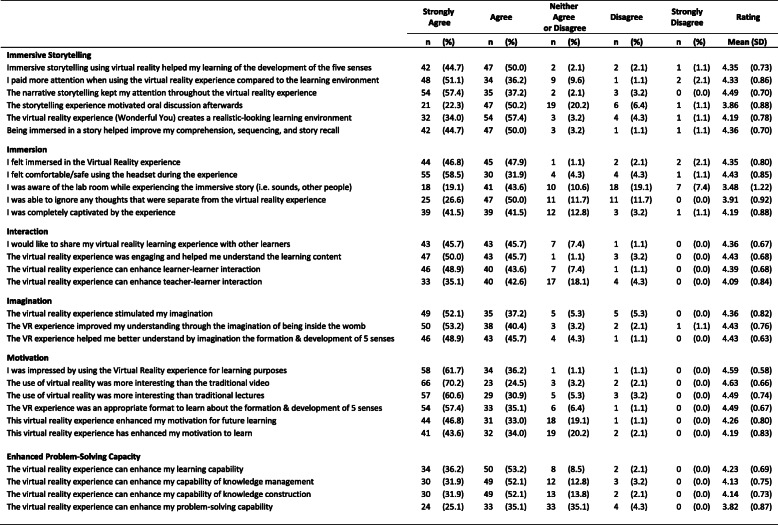
Percentage of scores in each of the 5 response categories (strongly agree to strongly disagree) for all items grouped under their respective subsections by all participants (*n* = 94). Final column contains the mean rating for each item

### Students perception of iVR storytelling

The opening questions on the evaluation questionnaire examined students’ perception of iVR storytelling as an active pedagogy. Overall, students reported a positive experience. The overall mean score was 4.26 (SD 0.61; range 1–5). 94.7% of respondents (*n* = 89) strongly agree or agree that the iVR storytelling experience helped their understanding of the five senses in the womb. 94.6% (*n* = 89) reported that the narrative element of the storytelling experience held their attention throughout the experience. A slightly smaller proportion of 72.5% (*n* = 68) agreed that the iVR storytelling experience motivated oral discussions afterwards. Moreover, 94.7% (*n* = 89) reported being immersed in a story helped improve their comprehension, sequencing, and story recall of the development of the five senses. These results indicate a meaningful educational experience and a high percentage of student satisfaction with this pedagogy.

### Student’s perception of levels of immersion, interaction, imagination, motivation, and enhanced problem-solving capacity during an experience with iVR storytelling

The remaining questions on the evaluation questionnaire examined students’ perception of levels of immersion, interaction, imagination, motivation, and enhanced problem-solving capacity during their experience with iVR storytelling. Students reported high levels of immersion, with an overall mean score of 4.07 (SD 0.61). 83% (*n* = 78) of participants noted being captivated by the experience. Students evaluation of the interaction of the iVR experience was also received positively with an overall mean score of 4.32 (SD 0.61). However, it must be noted a small percentage of students (22.4%, *n* = 21) responded neutrally or negatively that the iVR experience enhanced teacher-learner interaction, this will be examined in the discussion section of the paper. Students generally reported iVR experience engaged their imagination with an overall mean score of 4.40 (SD 0.63). 93.6%(*n* = 88) reported the imagination of being inside the womb improved their understanding and enabled their learning. Students rated high levels of motivation towards the iVR storytelling experience receiving an overall mean score of 4.40 (SD 0.63) with 97.9% (*n* = 92) reporting they were impressed with using iVR storytelling experience for learning purposes. 94.7% (*n* = 89) agreed that the iVR experience was more interesting than traditional videos. Students reported that the iVR experience enhanced their capability of knowledge management and construction of knowledge (84%, *n* = 79). However, a significant number of students (39.4%, *n* = 37) responded neutrally or negatively that the iVR experience enhanced their problem-solving capability. Overall participants self-reported perceptions of immersion, interaction, imagination, motivation, and enhanced problem-solving capacity with iVR storytelling was rated positively.

### Qualitative data analysis

The online questionnaire contained open-ended questions which allowed students to express their views and experience of using iVR: what they liked the most and what they least liked about using iVR storytelling.

Several topics were identified as significant themes including memorable learning experience, negative elements to iVR and the application of iVR in other areas of the Nursing and Midwifery curriculum. Some minor themes included previous use of VR and interest in purchasing a VR headset.

#### Memorable learning experience

The positive evaluations of the iVR storytelling experience were also evident in the narrative responses. Students acknowledged that the iVR experience immersed them into a story they have never or could ever experience in real life. Participants reported enjoying that the iVR scenario felt as if they themselves were in the womb, as one participant referred to the experience as *“real in an unreal setting”.* Others commented on how they felt immersed in the environment of the womb which they believed would help them remember in the future, compared to traditional teaching methods, and reinforced what they had previously been taught: *“I liked how it brought you into the environment and allowed you to be immersed in the development of the stages. The use of colour and animation really made it memorable.”, “It was captivating and made more sense to me because I saw it in real life rather than a diagram.”, “It held my attention more than a lecture”, “It was so realistic and added to my knowledge. Felt like I learnt a lot that I will remember”.*

Moreover, participants commented on how virtual reality felt “*fun*” and “*more interactive than normal”* compared to standard teaching methods. The immersion into the VR storytelling experience meant that participants had both an emotional and sensory experience, as well as a richer understanding of prenatal development: *“I felt submerged in the storytelling. It was amazing! Give me the chills!”*, *“Being able to focus completely and get a real-life sense of science.”*, *“I found it easier to understand the development of structures than the lectures”*, *“Experiencing ‘real-life’ as accurately as possible”*. Participants commented how using iVR storytelling as a teaching method removes distractions from their surrounding environment that can sometimes be present in a traditional classroom: *“That it takes you into a new environment and blocks out any distractions”.* Ultimately, this enabled participants to concentrate on the experience and increase their focus, as one participant appreciated “*being able to zone out from the room and focus on the video*”.

#### Negative elements to iVR

Conversely, participants described some negative aspects of the iVR experience. Several participants’ negative experiences were related to the headset, which they said was uncomfortable and claustrophobic to wear for the experience: *“The headset felt sweaty and heavy and gave me a sore neck”, “slightly heavy, would be uncomfortable for long periods of time”, “headset didn’t fit properly so had to readjust”.* Some participants reported feeling motion sickness and “*dizzy*” from using the headset and that their neck was sore from the turning involved in the experience. As previously mentioned, participants praised the experience for its lack of distractions, however, others mentioned they had a lot of noise distractions. Participants who used headphones for the experience were fully immersed, however students without headphones did get distracted by others: *“I could hear other peoples’ videos playing”*, *“not having earphones did not allow for thorough immersion as could hear other headsets.”*

Some students referred to technical difficulties relating to the required software and their smartphones. A common difficulty was with their hosting phone, rather than the VR equipment: *“Didn’t download, had to stream it” “phone wouldn’t download the app, Used staff members phone” “it would not play on aeroplane mode”*. Some mentioned that the headset was “*out of focus*” and the video had a “*blurry*” quality to it which was difficult to view. This was likely due to the adjustment setting on the headset before commencing the experience. Some participants were unable to download the app due to space capacity on their phones: *“low disk space on phone”, “no space”.* Issues arose regarding students who were unable or had not downloaded the app before the session: “*Wouldn’t show up in play store*”, “*due to the internet, it took a while to download”.*

#### Application of VR in nursing and midwifery education

Participants reported an overall positive attitude to including iVR in other aspects of their education and training. There were two key factors for participants who suggested the use of virtual reality in other modules. Firstly, it increased their concentration and ability to engage with the learning material; “*I was very attentive, more than lectures”, “I had a high level of concentration when using”, “It was interesting and held my attention”.* One participant noted that due to the lack of distractions while using the headset it “*makes you focus on one thing*”. Secondly, students felt it enabled them to visualise abstract concepts of anatomy and physiology that is central to their curriculum: “*You get a far better understanding of what the anatomy of the body looks like”, “Help to get a clearer picture of the topic”, “visualizing makes it easier to grasp the concept”.* Regarding the modules students would like to see virtual reality utilised in, there was a resounding appreciation of how it would be helpful in anatomy and physiology learning, where some participants specifying that it would help them understand cardiovascular, respiratory, gastrointestinal, central nervous and embryological concepts and the stages of labour. This potential usefulness of iVR is highlighted by its ability to allow students to interact, dissect and explore abstract anatomical objects enabling students to better understand spatial interpretation and depth perception of organs than traditional pedagogical approaches.

Minor themes emerging from the data included previous use of VR and purchasing a VR headset. Some participants referred to how they had previously used virtual reality through the medium of video games, at their own home or friends’ houses using gaming consoles. Some participants reported using during outdoor events such as roller coasters or tourist exhibitions. However, 65.95% (*n* = 62) participants reported they had never used VR before. Participants described that they would be interested in buying VR headset as they viewed it as an innovative and engaging way to learn: “*New and different way of learning*”, “*visual images and videos really help me learn*”, “*it makes learning abstract things easier if you can visualize it*”. However, other participants mentioned that they would not be interested in buying one as it did not align with their learning style: *“learn better by writing”,* “*Didn’t suit my way of learning*”. One participant reported that they would have equivalent experience by watching educational videos on YouTube: “*could just watch an informative YouTube video*”. Several participants described that they would be interested in buying a virtual reality headset, but it would be cost-dependent, as they were perceived to be expensive equipment: *“depends how much the device would cost”.* Some participants mentioned they would want to ensure its good quality and would prefer to purchase when more options become available.

### Observational data

Overall researchers observed students positively engaged with the iVR storytelling experience. Researchers noted an air of excitement amongst students, with most students turning up with the app downloaded and earphones ready to go. Students initially had many questions and appeared very enthusiastic e.g. “*will I really be inside a womb*?”

Researchers noted minor difficulties with the VR headsets. All participants were able to experience the iVR story; however, a few issues with set up and technical problems arose. Despite initial instruction, several students (*n* = 28) required a second demonstration on how to insert the phone into the VR headset one-to-one. Other minor issues included difficulty setting and adjusting the head strap on the VR headset. One researcher provided their phone to a participant as their smartphone did not have enough storage space to download the app.

Positive student interactions with the iVR storytelling experience were also witnessed. Researchers noted the external gaze point (i.e., what direction they were looking in: straight on or rotating in the chair/bed to see behind and around them) of the participants when engaging with the VR experience. Most students were observed moving their head and body looking up, down, left, right and behind them as though they were navigating through the iVR experience.

However, some students (*n* = 9) appeared less engaged and sat motionlessly. The researchers noted how students who wore headphones for the experience appeared more immersed and engaged in more head and body movement in following the iVR experience. Those not wearing headphones appeared distracted looking over in the direction of other students who also had no headphones as though they were distracted by their iVR sound. One participant was observed to be suffering from motion sickness, appeared pale but continued and adapted to the experience, after which they expressed, they really liked the experience and only felt slightly nauseous.

Members of the observational team noted differences in the degree of emotional and facial arousal according to the content being viewed. However, the research team was unable to determine what point of the VR experience the participant was viewing when this occurred. Some students were vocal in their reactions throughout the VR experience with comments such as “*oh my god, this is cool*”, “*this is weird*” and “*this is awesome*”.

## Discussion

This study aimed to provide a unique insight into Nursing and Midwifery students’ perception of the application of an iVR storytelling experience as an active pedagogy. By implementing “Wonderful You” (BHD Immersive), the authors set out to examine the students’ perceptions of iVR storytelling and its impact on their learning.

This study found immersive VR storytelling to be a positive and effective learning experience. iVR storytelling created an authentic learning experience comparable to the application of conventional storytelling [19] and digital storytelling [21] in Nursing and Midwifery education. Students reported that the storytelling narrative maintained their attention throughout and expressed that they had increased levels of engagement with the presented material compared to traditional learning environments. This may be related to the characteristics of iVR storytelling media used in which students are no longer passive viewers of a story but immersed in it as active participants. As such, VR users must actively discover the story, as the narrative and visuals elements evolve 360 degrees around them. These findings are consistent with Urstad et al. [32] who maintain that storytelling can capture a person’s attention and therefore generate strong emotional engagement. Similarly, Pieterse et al. [33] found that students felt more actively engaged with a 360 VR video experience than while watching a standard video. These results imply that iVR storytelling fosters high levels of attention and interest from students which are vital for active learning.

This study also aimed to examine student’s perception of levels of immersion, interaction, imagination, motivation, and enhanced problem-solving capacity with iVR storytelling. Overall high levels were reported by students in each category, particularly motivation, imagination and interaction which stimulated students desire to learn the educational content featured in the iVR storytelling experience. Students reported high levels of immersion; the head-mounted displays (HMD) blocked out visual distractions from the physical environment of the simulation laboratory, placing greater emphasis on the visual components of the story. Students who wore headphones further reported an even higher level of immersion as it blocked out any noise from surrounding students. These findings support previous research that found audio and visual features of IVR can elicit cognitive and affective responses from users that create a sense of immersion [34].

Similarly, high levels of interaction were reported by students stating that they would like to share their learning experience with others and that it could enhance learner-to-learner interaction. This may be as a result of the novel experience iVR storytelling provided students; however educational experiences that increase communication and collaboration amongst students have been shown to facilitate quality learning experience with significant positive outcomes for students [35]. A minority of students responded neutrally or negatively that the VR experience enhanced teacher-learner interaction. It should be noted that no debriefing session was held following this iVR storytelling experience. Debriefing has been identified as a critical factor to a successful experiential learning experience to promote student reflection and assimilate the learning experience, generating a deeper understanding. The omission of a debriefing session may have led to a decreased satisfaction for some with the level of teacher-learner interaction in this study [7, 36].

Rather than asking students to imagine themselves in a specific situation, VR perceptually immerses them in an experience, consequently triggering their imagination [37]. This study confirmed these findings with students acknowledging the iVR storytelling experience elicited their imaginations in believing they were inside a womb. Students further reported the imagination of being inside a womb improved their understanding as they were able to visualise the anatomy and enabled their learning of the formation of the five senses.

Evidence shows that Nursing and Midwifery students self-motivation plays a significant part in the learning process and if educators can connect learners with ideas and concepts that motivate them to discover more, positive learning outcomes are very likely to be achieved [38]**.** The greatest perceived gain from implementing iVR storytelling in the current study was an increase in students’ motivation to learn. Arguably students self-selected into the learning experience in the first instance so a subset of the self-motivated students in the class took part in the experience. Nonetheless, students indicated they were impressed with using iVR storytelling experience for learning purposes and felt iVR storytelling was an appropriate medium of learning the five senses and reported highly to the iVR experience motivating their future learning. As previously mentioned, students also found iVR storytelling provided greater motivation than traditional pedagogical approaches. Students could see the merits of iVR storytelling and would like to see it being used as a pedagogical tool in future learning particularly in areas of abstract concepts of anatomy and physiology that are central to their curriculum.

Students also reported high levels of enhanced problem-solving capacity following exposure to the iVR storytelling experience. For students to learn and gain a deep understanding of a concept, the content must be rendered meaningful to the learner [39]. When achieved, learning is transformed, and the student can make a connection with the material. This leads to the personal construction of knowledge, which in turn generates ownership of the new knowledge [40]. This speaks to constructivist theory for education in which learners construct knowledge through the process of making sense of the experience [41]. iVR helps students to conceptualise abstract knowledge and promotes spontaneous and creative elaboration on the topic, therefore improving student’s ability to examine new concepts and reasoning [22, 42]. This study supports these findings, as students expressed iVR storytelling experience improved their comprehension, sequencing and recall of abstract concepts such as anatomy which has been reported as being sometimes difficult to grasp when traditional pedagogical approaches are applied [23]. Previous research has highlighted students often struggle with comprehending complex concepts in biosciences due to a traditional pedagogical approach and the lack of hands-on student centered concrete activities [23, 24, 43]. VR offers a suitable medium and educational approach to potentially address this issue.

The third aim in this research study set out to examine the student’s engagement and interaction with the iVR storytelling experience. Observations are a critical component of developmental evaluation for new pedagogical approaches. Observations reinforced students’ self-reported positive learning experience. A clear sense of active engagement and participation with the iVR storytelling experience was observed, with most students expressing enthusiasm for the authentic learning experience. Herault et al. [44] also concluded that nursing students and faculty agreed immersive VR added value and increased engagement with their education from which the authors concluded that it was a great pedagogical approach to supplement traditional teaching practices. Active student interaction was observed throughout this study with clear signs of positive emotional and facial arousal noted by the research team. However, a small number of students appeared less engaged and remained motionless when viewing the iVR experience. It is possible that these students were unfamiliar with the concept of iVR and its ability to present images 360 degrees and were more accustomed to viewing a traditional film which requires you to look straight ahead. Furthermore, a small proportion of students expressed a negative evaluation of the iVR storytelling experience with students reporting physical discomfort from the VR headset and episodes of motion sickness. Suh & Prophet [34] presented similar findings citing immersive technology did not always create a positive learning experience and in the same way as this study caused motion sickness to a small proportion of users. Further research is required to determine what features of the iVR storytelling experience influenced the participants viewing behaviour and degree of emotional arousal, as well as specific triggers for motion sickness.

Several recommendations concerning the implementation of iVR storytelling as an active pedagogy can be made from the finding of this pilot study. Results from the observations exemplify the importance of determining the student’s role in the iVR experience and communicating this to them before the iVR experience commences, so that students will be aware of what is expected of them. The adaptation of The International Nursing Association for Clinical Simulation and Learning (INACSL) [45] standards of best practice for simulation could provide a framework to structure the iVR storytelling experience and assist in the organisation of the experience. A co-ordinated pre-briefing session in which the student role and objectives are communicated, as well as a debriefing session in which the students reflect and summarise their learning experience is recommended. Minor set-up issues were easily managed by the research team as students became accustomed to using the VR headsets. However, the authors recommend familiarising both faculty and students with the VR headset before an educational experience. Given the fact that many students may not be accustomed to using HMD technology, it is crucial to provide a single-focused and engaging viewing experience to avoid cognitive overload in students. The application of a VR headsets with built-in headphones or capacity for Bluetooth is recommended to immerse the student into the VR experience further and avoid distraction for other students when using VR in a small group teaching setting. The research team advocates for easier access to different directional gaze points by use of a swivel chair with reclining capability, as opposed to the stationary or non-rotational chairs and beds utilised in the current study. Finally, in order for the application of iVR storytelling to be integrated into Nursing and Midwifery programmes it is important to engage course coordinators in trialling a VR application to achieve interest in and engagement with the value in its application in the various curricula.

### Limitations

The authors note that the findings of this study are limited to a moderate scale evaluation study based on a convenience sample from a single site that relied on students self-reporting. Therefore, these results are not generalisable to the wider Nursing and Midwifery student population. This study lacked the implementation of a best practice simulation framework such as INACSL [45], therefore the effectiveness of the iVR storytelling experience could have been further enhanced by integrating best practices such as a guided debriefing session. Notwithstanding these limitations, this work offers valuable insights into students’ perspective of the application of immersive VR storytelling as a pedagogical approach to Nursing and Midwifery education. The application of iVR storytelling for Nursing and Midwifery education will only progress as researchers continue to experiment and share what they observed to be effective and ineffective before the full potential of this innovative medium as an active pedagogy can be understood or realised.

## Conclusions

This exploratory pilot study addresses the gap in the literature examining the application of immersive VR storytelling as an active pedagogy in Nursing and Midwifery education. This study identified attributes of iVR storytelling such as immersion, interaction and imagination promote student’s motivation and problem-solving capacities, which in turn creates a meaningful and memorable learning experience for students. iVR storytelling can provide a valuable opportunity to imagine a conceptually complex idea, increasing the possibility of a deeper understanding of concepts that might not be easily understood. The full potential of this new medium of iVR storytelling has yet to be seen. Continued technological advancements and investigation into iVR storytelling within Nursing and Midwifery education may provide new insights and future possible applications.

## Supplementary information

**Additional file 1.**

**Additional file 2.**

## Data Availability

The datasets used and/or analysed during the current study are available from the corresponding author on reasonable request.

## Abbreviations

VR: Virtual Reality
HMD: Head Mounted Display
iVR: Immersive Virtual Reality
VRLE: Virtual Reality Learning Environments
